# Changes in Exposure to Secondhand Smoke Among Youth in Nebraska, 2002–2006

**Published:** 2008-06-15

**Authors:** Lazarous Mbulo

**Affiliations:** Nebraska Department of Health and Human Services, Division of Health Promotion and Disease Prevention, Tobacco Free Nebraska

## Abstract

**Introduction:**

Secondhand smoke is a major cause of morbidity and mortality. It has been associated with serious health problems in both children and adults. Efforts to reduce exposure to secondhand smoke in Nebraska have included programs to prevent tobacco use among young people and campaigns for smoke-free workplaces and homes. Despite these interventions, young people continue to be exposed to secondhand smoke at an unacceptably high rate. The objective of this study was to examine the extent to which Nebraska public middle and high school students were exposed to secondhand smoke in 2002 and 2006, to evaluate factors associated with this exposure, and to propose interventions.

**Methods:**

The Nebraska Youth Tobacco Survey was administered in 2002 and 2006 to a representative sample of students from public middle and high schools. All students who chose to participate completed an anonymous, self-administered survey that included questions on demographics, tobacco use, tobacco-related knowledge and attitudes, and exposure to secondhand smoke. Data were weighted to account for nonresponses at both student and school levels and to ensure generalizability of the estimates for public school students in Nebraska according to their grade, sex, and race/ethnicity. This study analyzed a subset of responses on secondhand smoke exposure, which was defined as being in a room or vehicle during the previous 7 days with someone who was smoking cigarettes.

**Results:**

Secondhand smoke exposure in a room, a vehicle, or both declined significantly among all students from 2002 (69.0%) to 2006 (61.3%). In both 2002 and 2006, students were significantly more likely to be exposed to secondhand smoke in a room than in a vehicle (64.4% vs 48.2% in 2002 and 56.9% vs 40.2% in 2006). Among racial and ethnic groups, only white students experienced a significant decline in exposure from 2002 (70.0%) to 2006 (61.4%). Girls were significantly more likely to be exposed to secondhand smoke in 2006 than were boys, and only boys experienced a significant overall decline in exposure from 2002 (69.3%) to 2006 (57.7%). Smoking behaviors and attitudes continued to influence secondhand smoke exposure from 2002 to 2006, although students experienced significant declines whether they were smokers or nonsmokers, and whether they lived with a smoker or not. Those with close friends who smoked and those who did not perceive secondhand smoke as harmful, however, did not benefit.

**Conclusion:**

These data indicate reductions in exposure to secondhand smoke among Nebraska's middle and high school students, but exposure remains a problem, particularly in rooms. Adoption of a comprehensive statewide smoke-free policy will contribute to significantly reduced exposure to secondhand smoke among young people in public places, but other measures to address exposure in the home and private vehicles are needed or should be strengthened. These include physician counseling based on behavioral change theory to encourage cessation and home-based no-smoking rules, in addition to interventions that target minorities, who are disproportionately affected by secondhand smoke exposure. Evaluation of existing measures, such as programs to prevent tobacco use among young people and campaigns to collect pledges for smoke-free homes, will be required to determine their effectiveness in reducing exposure to secondhand smoke among youth in Nebraska.

## Introduction

Tobacco use contributes to diseases and deaths among users and nonusers and remains a major public health challenge. Increasing research evidence shows that exposure to secondhand smoke is a major cause of morbidity among nonsmokers, including children (1). Secondhand smoke is a combination of smoke exhaled by smokers and the sidestream smoke from the end of a burning cigarette. It contains more than 4000 chemicals, some of which cause cancer (2). Among children, exposure to secondhand smoke worsens asthma, slows lung growth, and increases the risk for sudden infant death syndrome, acute respiratory infections, and ear infections (1). In adults, exposure to secondhand smoke has immediate adverse effects on the cardiovascular system and causes coronary heart disease, lung cancer, and other health complications (1).

Despite major health problems associated with tobacco use, more than one-fifth of adults in Nebraska (21.3%) continue to smoke cigarettes (3). Adults who smoke are more likely to expose young people who live with them to secondhand smoke unless they voluntarily establish and comply with smoke-free rules in their homes (1).

An effective strategy to reduce exposure to secondhand smoke is to implement smoke-free policies in public places such as bars, restaurants, and other workplaces; increasingly, societies are adopting such policies (1). Where these policies are adopted, exposure to secondhand smoke among adults and youth is significantly lower in public places (1) but not necessarily in homes and family vehicles, given that smoke-free statutes and policies do not cover these private environments. Ensuring protection against secondhand smoke in private homes and vehicles is a domain of individuals. However, adoption of smoke-free policies for public places can influence individuals to adopt smoke-free rules for their homes to protect their family members from secondhand smoke (4).

The Nebraska Department of Health and Human Services, through its Tobacco Free Nebraska program (www.hhs.state.ne.us/tfn/), designs and implements tobacco control and prevention interventions. For 3 years starting in fiscal year 2001, the department received $7 million annually as a result of the Master Settlement Agreement between states and tobacco companies (5). One of the goals of Tobacco Free Nebraska is to reduce exposure to secondhand smoke among Nebraskans. Its No Limits movement targets young people in particular, by engaging them in activism and peer education. However, Nebraska has not returned to the $7 million funding level since 2003; after budgeting only $410,000 in fiscal year 2004, the state allocated $2.5 million in fiscal year 2005 and $3 million in fiscal year 2006, including Master Settlement funds (5).

In Nebraska, Lincoln was the only major city with a comprehensive smoke-free policy in place during 2002 to 2006; it took effect in 2005, following the efforts of Tobacco Free Nebraska and the local coalition, along with other partners  (6). However, the state legislature introduced a comprehensive smoke-free law in 2007 and passed it in February 2008 (7). By that time, local antitobacco coalitions and other health groups in the state had made considerable progress in educating the public about the dangers of secondhand smoke (8). Among their strategies were efforts to encourage people to adopt voluntary smoke-free policies in their homes, such as distributing "no smoking inside the house" door plaques and smoke-free homes pledges, and sponsoring legal measures to reduce exposure to secondhand smoke in apartment complexes (www.omahasmokefreeapartments.info/12.html).

The objective of this study was threefold: to report secondhand smoke exposure among Nebraska public middle and high school students surveyed in 2002 and 2006, to analyze which groups of students remain at high risk of exposure, and to propose evidence-based interventions to address this risk.

## Methods

### Sampling

The Youth Tobacco Survey, developed by the Centers for Disease Control and Prevention (CDC), is a tool for designing, implementing, and evaluating tobacco use prevention and control programs. The Nebraska Department of Health and Human Services administers the survey and CDC's Office on Smoking and Health provides technical assistance in data collection.

The survey does not require institutional review board approval. However, Nebraska's Department of Health and Human Services works with the state's Department of Education and the schools to collect the data. Schools and students are free to decide whether they want to participate, and schools require parental consent for student participation.

The Nebraska Youth Tobacco Survey was conducted in spring 2002 and 2006. The survey is a 2-stage cluster-sample design aimed at gathering a representative sample of middle and high school students from Nebraska public schools. The sampling units were public middle schools (grades 6–8) and high schools (grades 9–12). In the first sampling stage, schools were stratified into middle and high schools, and 2 samples were drawn from each stratum. Schools were selected for participation in the survey with a probability proportional to the number of students enrolled. The second sampling stage consisted of systematic equal probability sampling (with a random start) of classes from each school that participated in the survey. All second period classes in the selected schools were included in the sampling frame.

All students in the selected classes, regardless of whether they used tobacco, were eligible to participate in the survey. Students completed an anonymous, self-administered questionnaire. Data were weighted to account for nonresponses at both student and school levels and to ensure generalizability of the estimates for public school students in Nebraska according to their grade, sex, and race/ethnicity.

### Measures

The Youth Tobacco Survey assesses exposure to secondhand smoke in public and private spaces with 2 questions: 1)  "During the past 7 days, on how many days did you ride in a car with someone who was smoking cigarettes?" and 2) "During the past 7 days, on how many days were you in the same room with someone who was smoking cigarettes?" Answers to both questions are measured on a 5-point scale. In addition to questions on exposure to secondhand smoke, the survey asks questions about smoking status and history, living with a smoker, smoking status of friends, attitudes toward secondhand smoke, and demographics. This study analyzes secondhand smoke exposure in terms of these other characteristics. SUDAAN software release 9 (Research Triangle Institute, Research Triangle Park, North Carolina) was used to calculate prevalence estimates and confidence intervals. Significance was set at *P* < .05.

## Results

In 2002, a total of 2944 students in 46 middle schools and 2677 students in 41 high schools participated in the survey. In 2006, a total of 2295 students in 41 middle schools and 2924 students in 62 high schools participated in the survey. The overall response rate, a product of the school and individual student response rates, was 76.2% for both middle and high schools in 2002 and 65.5% in 2006. Student response rates were calculated based on the number of students who participated in the survey, regardless of whether they answered all questions. Less than 5% of students had missing responses on the 2 secondhand smoke exposure questions, and less than 5% of students who answered those 2 had missing responses on other questions.

The 2002 and 2006 current smoking prevalence rates among Nebraska high school and middle school students were determined by the question, "During the past 30 days, on how many days did you smoke cigarettes?" Smoking prevalence was significantly higher among high school students in 2002 (28.2%) than in 2006 (19.6%) and was not significantly different among middle school students from 2002 (7.0%) to 2006 (5.3%). Neither survey found any significant difference between high school girls and boys or between middle school girls and boys on current smoking prevalence.

The Table shows the results of the analysis on exposure to secondhand smoke. Overall, the proportion of students who were exposed to secondhand smoke in a room, vehicle, or both declined significantly from 2002 (69.0%) to 2006 (61.3%). In both 2002 and 2006, students were significantly more likely to be exposed to secondhand smoke in a room than in a vehicle (64.4% vs 48.2% in 2002 and 56.9% vs 40.2% in 2006, data not shown).

Among racial and ethnic groups, only white students experienced a significant decline in exposure from 2002 (70.0%) to 2006 (61.4%). Exposure to secondhand smoke was significantly higher among high school students than among middle school students in both survey years; nevertheless high school students experienced a significant decline in exposure from 2002 (74.0%) to 2006 (64.4%).

Although more than two-thirds of both girls and boys were exposed to secondhand smoke in a room, a vehicle, or both in 2002, girls were significantly more likely than boys to be exposed in 2006 (64.9% vs 57.7%). Furthermore, only boys experienced a significant decline in exposure to secondhand smoke from 2002 (69.3%) to 2006 (57.7%).

Students who smoked were significantly more likely than nonsmokers to be exposed to secondhand smoke. More than 90% of smokers were exposed to secondhand smoke in both 2002 and 2006 compared with less than 65% of nonsmokers during the same periods. However, both smokers and nonsmokers experienced a significant decline in exposure to secondhand smoke from 2002 to 2006 (95.1% to 90.5% vs 62.5% to 56.2%, respectively).

Students who ever tried smoking cigarettes were significantly more likely to report exposure to secondhand smoke than were those who never tried smoking a cigarette in both 2002 and 2006, and both groups experienced a significant overall decline from 2002 to 2006 (84.2% to 78.9% and 56.1% to 50.1%, respectively).

In both 2002 and 2006, students who had close friends who smoked were significantly more likely to be exposed to secondhand smoke than were students who had no close friends who smoked. Only students with no close friends who smoked experienced a significant decline in exposure to secondhand smoke from 2002 (57.1%) to 2006 (50.4%).

In both survey years, students who lived with a smoker were significantly more likely to be exposed to secondhand smoke than were those who did not, although both groups experienced a significant decline in exposure from 2002 to 2006 (89.6% to 83.9% and 55.4% to 46.7%, respectively).

Students who perceived exposure to secondhand smoke as being harmful to their health were significantly less likely to report exposure to secondhand smoke than were those who did not perceive secondhand smoke as harmful. Furthermore, only students who perceived secondhand smoke as harmful experienced a significant decline in exposure from 2002 (66.3%) to 2006 (57.9%).

## Discussion

This analysis of responses to the 2002 and 2006 Nebraska Youth Tobacco Survey shows that most student groups have experienced significant declines in their exposure to secondhand smoke. Nevertheless, students who were current or former smokers, or had ever tried smoking, and those who lived with smokers or had close friends who smoked, remained at high risk of secondhand smoke exposure in 2006, even when their exposure declined significantly from 2002.

One of the major goals of the Nebraska Department of Health and Human Services Tobacco Free Nebraska program is to reduce exposure to secondhand smoke. Despite declines in funding over time, the program has continued its effort to reduce secondhand smoke exposure in the state through ongoing interventions. Although this study does not measure the connection between these interventions and exposure to secondhand smoke, it does examine the exposure to secondhand smoke among youth in the years following an injection of funding to Tobacco Free Nebraska (5) and during a period when ordinances prohibiting smoking in public buildings were being widely adopted nationally and in the city of Lincoln.

In this climate, Nebraska students reported a significant decline in exposure to secondhand smoke from 2002 to 2006; nevertheless, in 2006, 61.3% of Nebraska students surveyed had been exposed to secondhand smoke in a room, a vehicle, or both. In particular, well over half of the students in both years had been exposed to secondhand smoke in a room, significantly more than in a vehicle. The Youth Tobacco Survey questionnaire does not specify what "room" means, but for this population homes are implied, particularly since almost all schools (92.7%) in Nebraska have comprehensive smoke-free policies for school buildings (3). Rooms may also include places such as restaurants, since the state had no comprehensive smoke-free law covering public places during the study period. However, the home remains the primary source of exposure to secondhand smoke for infants and children and a major source of secondhand exposure for nonsmoking adults (1). The results thus suggest the need to focus on homes for smoke-free interventions to reduce exposure of youth to secondhand smoke.

Reductions in exposure to secondhand smoke among racial and ethnic groups from 2002 to 2006 were only significant among the white students, reflecting ongoing disparities in tobacco use and health outcomes. The lack of change among the other racial and ethnic groups (which are minorities in the state) suggests that interventions should target these populations.

Although both girls and boys were equally likely to be exposed to secondhand smoke overall, only boys experienced a significant decline in exposure from 2002 to 2006. Furthermore, girls were significantly more likely than boys to be exposed in a room in 2006. If these sex differences are confirmed elsewhere, they warrant further analysis.

Students who smoke and spend time with smokers, and those who do not perceive secondhand smoke as a health threat, could benefit from a comprehensive approach that targets normative and behavioral change in terms of both secondhand smoke and smoking behavior. These strategies should target both young people and adults in the state.

Tobacco Free Nebraska coordinates the efforts to prevent tobacco use for the state health department, local coalitions, and other health organizations. The program aims to eliminate exposure to secondhand smoke, promote cessation through the Nebraska Tobacco Quitline, and reduce tobacco use among youth. Specifically, its No Limits program is a youth-led movement that uses education and activism as key strategies to empower young people not to use tobacco (www.nolimitsnebraska.com/).

In addition to Tobacco Free Nebraska, local coalitions and other health advocates such as the American Cancer Society, American Heart Association, and American Lung Association were ultimately successful in promoting the passage of a comprehensive statewide smoke-free law in 2008 (7). This will be a major step toward reducing exposure to secondhand smoke in public places. A policy approach that prevents exposure to secondhand smoke in public places, including worksites such as bars and restaurants, has the potential to change social norms (1,9,10). Workers who are protected by smoke-free policy may be more likely to want their children and spouses also to be protected from secondhand smoke (1,9,10). Until this state law was passed, only Lincoln had a comprehensive smoke-free law.

A comprehensive smoke-free policy helps smokers to consider quitting or reducing the number of cigarettes smoked (11,12). The Nebraska Quitline, as part of the overall tobacco prevention program in Nebraska, is an important service for smokers who want to quit (13). As smokers quit, the potential for youth to be exposed in homes would also be reduced.

At individual and community levels, adoption of smoke-free rules in both homes and vehicles is an important step in reducing young people's exposure (14). The home is an appropriate focus area, since young people are prone to be exposed to secondhand smoke in homes. The high exposure to secondhand smoke in a room found in the Youth Tobacco Survey (56.9% in 2006) occurs in the context of a 21.3% smoking prevalence among adults in Nebraska (3). Thus, 1 adult smoker is likely to affect more than 1 child. Addressing exposure to secondhand smoke in homes therefore can disproportionately reduce exposure to secondhand smoke among young people.

In addition to supporting a statewide comprehensive smoke-free law, Tobacco Free Nebraska worked with local coalitions to target secondhand smoke in homes and vehicles (www.nlc.state.ne.us/epubs/H8250/B009-2003.pdf) with community grants from the Master Settlement Agreement (15) and technical support to local coalitions. For example, in 2001–2003, one of the funded coalitions was Buffalo County Tobacco Free, which designed and implemented a 5-year action plan targeting exposure to secondhand smoke among teenagers (5). The coalition educated Buffalo County residents about the harmful effects of secondhand smoke on children's health and collected pledges from adults vowing not to smoke or allow others to smoke around their children as part of the Smoke-Free County Challenge, sponsored by the National Association of Counties. In a separate intervention, the Indian Center, Inc, in Lincoln successfully recruited 47 American Indian households to sign a "smoke-free household" proclamation with no-smoking rules for minors and no smoking in the home for adults (5).

Although Tobacco Free Nebraska and local coalitions are working to reduce exposure to secondhand smoke across the state and in their communities, there is a need to reexamine the strategies in place. Gehrman and Hovell (16) suggest interventions based on behavior change theory that combine physician counseling and home-based approaches, including cessation, to reduce exposure to secondhand smoke. Behavior change theory instills practitioners with concrete skills and strategies to help them foster their self-efficacy and emphasizes ongoing reinforcement for positive behavior changes. For example, physician counseling might involve giving mothers skills to confront their husbands who come home with friends who smoke (16).

Health promotion and antitobacco groups in Nebraska could also learn from states in which public housing authorities and private apartment owners and renters have taken steps to make their apartments smoke-free (16,17). Utah's "nuisance law," for example, has been applied to the issue of secondhand smoke drift in condominiums. Nuisance has been defined by statute to include secondhand smoke that drifts into a condominium more than once in each of 2 or more consecutive 7-day periods (18). To guide landlords and tenants in understanding and implementing these changes, Utah's Tobacco Prevention and Control program has created comprehensive Internet resources (18).

Some of Nebraska's local antitobacco coalitions target minority populations in their interventions, for example, by using Spanish in their media campaigns. These efforts could benefit from a component that includes intensive group-specific education that emphasizes the dangers of secondhand smoke and encourages adoption of smoke-free rules in homes. Strategies such as physician intervention, counseling for parents, cessation promotion, and behavior change reinforcement through the media (16) should focus on minority populations. This may require getting more minorities involved in local coalition activities.

Based on the results of the Youth Tobacco Survey in both 2002 and 2006, reducing tobacco use among youth, including changing perceptions about secondhand smoke, would be an important component of future youth-oriented risk reduction efforts. Although smoking rates among youth have been declining since the late 1990s in Nebraska, as throughout the United States, the trend is now leveling off (15,19). Reducing tobacco use among youth will require targeting this group with media messages about the dangers of tobacco, continuing to enforce restrictions of tobacco sales to minors, and empowering young people to recognize and resist the marketing tactics of tobacco companies (20).

This study has several limitations. First, the data represent only students in public middle and high schools in Nebraska. Second, the data were collected through a self-reported and anonymous survey; thus, responses cannot be validated. Finally, reporting secondhand smoke exposure "in a room" is not specific enough.

### Conclusions

Nebraska has had a comprehensive tobacco control and prevention program since 2000. Although the program's funds were cut in 2004, Youth Tobacco Survey data indicate reductions in secondhand smoke exposure among middle and high school students. However, exposure to secondhand smoke in this age group remains a problem. The newly passed statewide smoke-free legislation marks a substantial gain in the effort to reduce exposure to secondhand smoke among youth in public places, but other measures to reduce exposure in the home and private vehicles are needed or should be strengthened. These include physician counseling based on behavioral change theory to encourage cessation and home-based no-smoking rules among adults. In addition, measures adopted should focus on minority populations, which in this study did not show significant decline in exposure to secondhand smoke. Existing measures such as campaigns to get pledges for smoke-free homes and programs to prevent tobacco use among youth, including programs that focus on minority populations, should be evaluated to determine their effectiveness.

Research is needed to examine the effect of the different statewide public health media activities on raising awareness about the dangers of secondhand smoke and of local antitobacco coalition activities. In addition, future surveys should ask about exposure to secondhand smoke in homes rather than exposure in a room. Furthermore, it may be useful to examine the exposure to secondhand smoke among girls, who have not had the same degree of reduction in exposure as boys, especially in rooms.

## Figures and Tables

**Table.** Prevalence of Exposure to Secondhand Smoke Among Middle and High School Students^a^, Nebraska Youth Tobacco Survey, 2002 and 2006

**Characteristic**	2002		2006	

	n	% Exposed (95% CI)	n	% Exposed (95% CI)
**All students^b^**	5341	69.0 (66.5-71.4)	5076	61.3 (59.1-63.4)^c^
**Race/ethnicity**				
White	4329	70.0 (67.2-72.7)	3822	61.4 (59.0-63.6)^c^
African American	338	64.1 (60.3-67.6)	452	63.0 (56.6-68.9)
Hispanic	312	57.1 (50.6-63.3)	441	56.4 (50.0-62.5)
Native American	148	82.0 (73.5-88.2)	137	76.0 (67.1-83.1)
**School level**				
Middle school	2698	62.0 (59.1-64.8)	2208	56.8 (53.4-60.1)
High school	2643	74.0 (71.4-76.5)	2868	64.4 (62.0-66.8)^c^
**Sex**				
Girls	2805	68.9 (66.0-71.7)	2603	64.9 (62.4-67.4)
Boys	2519	69.3 (66.5-72.0)	2457	57.7 (54.8-60.6)^c^
**Smoking status**				
Current smoker^d^	905	95.1 (93.4-96.4)	647	90.5 (87.7-92.7)^c^
Nonsmoker	4261	62.5 (60.3-71.2)	4207	56.2 (54.0-58.4)^c^
**Ever tried cigarette smoking**				
Yes	2312	84.2 (81.6-86.4)	1827	78.9 (76.6-81.0)^c^
No	2697	56.1 (53.7-58.5)	2859	50.1 (47.6-52.6)^c^
**Close friend(s) smoke**				
Yes	1815	85.0 (82.7-87.1)	1511	81.0 (78.5-83.3)
No	3041	57.1 (54.8-59.4)	3110	50.4 (48.1-52.8)^c^
**Live with**				
Smoker(s)	2088	89.6 (87.9-91.1)	1923	83.9 (81.5-86.0)^c^
Nonsmoker(s)	3166	55.4 (52.1-58.6)	3050	46.7 (43.9-49.5)^c^
**Perceive secondhand smoke as harmful to health**				
Yes	4102	66.3 (63.3-68.7)	3953	57.9 (55.7-60.2)^c^
No	1093	77.3 (73.5-80.7)	1091	73.5 (70.4-76.4)

a Survey respondents who indicated that they had been exposed to secondhand smoke in a room, in a car, or both during the 7 days before being surveyed.

b The total number in each category (e.g., sex, smoking status) does not always add up to the total number "All Students" because cases with missing data on the category being analyzed were not used in the analysis.

c Indicates significant change (*P* < .05) from 2002.

d Current smoker is defined as a person who smoked cigarettes on 1 or more of the 30 days before being surveyed.
